# Gene expression analysis of human prostate cell lines with and without tumor metastasis suppressor CD82

**DOI:** 10.1186/s12885-020-07675-7

**Published:** 2020-12-09

**Authors:** Pushpaja Dodla, Vanitha Bhoopalan, Sok Kean Khoo, Cindy Miranti, Suganthi Sridhar

**Affiliations:** 1 Department of Cell and Molecular Biology, Grand Valley State University, Allendale, MI 49401 USA; 2 Department of Cellular and Molecular Medicine, University of Arizona Cancer Center, University of Arizona, Tucson, AZ 85724 USA; 3 Department of Integrative Biology, University of South Florida, 140, 7Th Avenue S, University of South Florida, St. Petersburg, FL 33701 USA

**Keywords:** CD82, KAI1, Metastasis tumor suppressor, Prostate cancer, Microarray, Gene expression

## Abstract

**Background:**

Tetraspanin CD82 is a tumor metastasis suppressor that is known to down regulate in various metastatic cancers. However, the exact mechanism by which CD82 prevents cancer metastasis is unclear. This study aims to identify genes that are regulated by CD82 in human prostate cell lines.

**Methods:**

We used whole human genome microarray to obtain gene expression profiles in a normal prostate epithelial cell line that expressed CD82 (PrEC-31) and a metastatic prostate cell line that does not express CD82 (PC3). Then, siRNA silencing was used to knock down CD82 expression in PrEC-31 while CD82 was re-expressed in PC3 to acquire differentially-expressed genes in the respective cell line.

**Results:**

Differentially-expressed genes with a *P* < 0.05 were identified in 3 data sets: PrEC-31 (+CD82) vs PrEC-31(−CD82), PC3–57 (+CD82) vs. PC3-5 V (−CD82), and PC3–29 (+CD82) vs. PC3-5 V (−CD82). Top 25 gene lists did not show overlap within the data sets, except (CALB1) the calcium binding protein calbindin 1 which was significantly up-regulated (2.8 log fold change) in PrEC-31 and PC3–29 cells that expressed CD82. Other most significantly up-regulated genes included serine peptidase inhibitor kazal type 1 (SPINK1) and polypeptide N-acetyl galactosaminyl transferase 14 (GALNT14) and most down-regulated genes included C-X-C motif chemokine ligand 14 (CXCL14), urotensin 2 (UTS2D), and fibroblast growth factor 13 (FGF13). Pathways related with cell proliferation and angiogenesis, migration and invasion, cell death, cell cycle, signal transduction, and metabolism were highly enriched in cells that lack CD82 expression. Expression of two mutually inclusive genes in top 100 gene lists of all data sets, runt-related transcription factor (RUNX3) and trefoil factor 3 (TFF3), could be validated with qRT-PCR.

**Conclusion:**

Identification of genes and pathways regulated by CD82 in this study may provide additional insights into the role that CD82 plays in prostate tumor progression and metastasis, as well as identify potential targets for therapeutic intervention.

**Supplementary Information:**

The online version contains supplementary material available at 10.1186/s12885-020-07675-7.

## Background

Metastasis, the spread of malignant cells from a primary tumor to surrounding tissues and distant organs involves complex cell signalling processes and regulators. Despite the ongoing research and therapeutic development, metastatic cancer remains incurable. The survival rates for metastatic cancers vary, but at large, are extremely low. If metastatic regulators and the cell signaling processes governing metastasis are identified and fully elucidated, they can be potential targets for oncologic treatment.

Prostate cancer is the second leading cause of cancer death in males in the United States. Metastatic prostate cancer has a five-year survival rate of 31%. Although many genes involved in prostate tumor development have been identified, the exact role that they play in tumor progression or metastasis is unclear. CD82, a protein product of the *KAI-1* or *CD82* gene, was first identified as a metastasis tumor suppressor in rat prostate cells in 1995 [1]. Since then, CD82 expression levels have been reported to be negatively correlated to the metastatic potential in prostate tumors [2–4] and other epithelial tumors including gastric [5], colon [6, 7], cervix [8, 9], breast [10, 11], skin [12], bladder [13, 14] lung [15], pancreas [16], liver [17–19], and thyroid [20]. CD82 currently serves as a diagnostic biomarker and its down-regulation is recognized widely as a predictor of metastatic potential in various solid malignant tumors [21].

CD82 is a member of the tetraspanins, which is a family of proteins with 4 transmembrane domains: one large and one small extracellular loop and two short cytoplasmic N- and C-domains; the large extracellular loop has at least two disulfide bonds [22, 23]. Tetraspanins play a major role in cell proliferation, adhesion, motility, signaling, and metastasis [21–24].. CD82 is known to associate with integral protein such as integrins (α3β1, α4β1, α5β1, α6β1, and αvβ2), cell adhesion molecules (E-cadherin, EWI-2), growth factor receptors such as epidermal growth factor receptor (EGFR), other tetraspanins (CD9, CD81, CD151), and intracellular signaling molecules such as protein kinase C [25–28]. CD82 has been well documented as an inhibitor of cell motility, invasion, and survival in cancer cells [25–27], with varied inhibition mechanisms. For example, CD82 regulation involves EGFR, hepatocyte growth factor receptor (c-Met), and transforming growth factor beta (TGF-β) in breast, prostate, and kidney cancers, respectively [29–31]. In breast cancer cells, CD82 inhibits ligand-induced dimerization of EGFR, attenuating the downstream signalling pathways of mitogen-activated protein kinase (MAPK), signal transducer and activator of transcription protein (STAT), and mammalian target of rapamycin (mTOR) that leads to cell proliferation and survival [29, 32]. CD82 also regulates EGFR ubiquitylation by recruiting protein kinase C and phosphorylating both EGFR and EGFR ubiquitin ligase E3 (Cbl) to promote internalization of EGFR [33, 34]. In metastatic prostate cell line PC3, restoration of CD82 suppressed integrin-mediated activation of c-Met, leading to decreased activation of a protooncogene tyrosine kinase (Src) and subsequent deactivation of several Src substrates, including breast cancer anti-estrogen resistance 1 Cas family member (p130Cas), focal adhesion kinase (FAK) [30], and p130Cas-Crk (an adapter protein) coupling and deactivation of CUB domain containing protein 1 (CDCP1) [35]. The exact mechanism by which CD82 inhibits Src is unclear, but it is not through inhibition of the receptor c-Met upstream [30]. c-Met inhibition by CD82 could involve mechanisms similar to those observed in breast cancer cells. A recent study suggests that CD82 may suppress epithelial to mesenchymal transition (EMT) in prostate cancer cells on fibronectin matrix by laterally interacting with α3β1 and α5β1 integrins to repress integrin signaling [36], inhibiting cell migration and invasion. In renal carcinoma cells, CD82 seems to play a prominent role in migration and invasion by blocking TGF-β1/Smad signaling pathway. When CD82 was overexpressed in these cells, the expression of both metalloproteinases MMP2 and MMP9 and TGF-β1 protein, a regulator of MMPs, were significantly decreased. CD82 overexpression also affected the phosphorylation state of the transcription factors Smad2 and Smad3, the downstream signaling molecules to TGF-β1 [31].

On the other hand, CD82’s role as a positive regulator has been extensively studied in T cell signaling. CD82 promotes T cell receptor signaling by triggering actin polymerization and stabilizing the downstream signaling of the T cell receptor (TCR/CD3) [37, 38]. CD82 promotes changes in T cell morphology involving the Rho GTPase pathway (Rho A, Rac1, and Cdc42) and through association with the guanine nucleotide exchange factor Vav1 and the adapter molecule SLP76 [39]. When T cells interact with antigen-presenting cells, a dynamic re-localization of CD82 and F-actin was observed at the periphery of the immune synapse, suggesting CD82’s role in membrane dynamics during T-cell signaling [40]. CD82 has been shown to promote enhanced cell to cell adhesion through E-cadherin in epithelial cells, i.e., CD82 expression in prostate cancer cells promoted E-cadherin-induced adhesion strongly by stabilizing E-cadherin’s association with β-catenin, a complex required for E-cadherin function and stability [41].

Studies on hematopoietic stem cells (HSC) have revealed CD82 positively regulates both in vivo and in vitro homing of HSC to the bone marrow for bone marrow engraftment [42, 43]. When CD82 was knocked down in mice, CD82 null mice had reduced long-lived HSC in vivo, with a decreased affinity of the cells to the endosteum. In addition, CD82 null mice exhibited weaker and smaller bones along with a decreased number of osteoclasts, increased adipogenesis, and decreased bone formation rate overall. In vitro phenotypes exhibited increased adipocyte numbers, defective osteoclast polarization, and decreased osteoblast differentiation and mineralization, indicative of defects in osteoclast, osteoblast, and adipocyte lineages [44]. Furthermore, a conditional CD82 knockout study in osteoclasts revealed CD82 loss did not affect cortical bone, osteoblasts or adipocytes [45]. However, an increase in osteoclast function and defects in actin assembly with altered osteoclast morphology were observed. The expression of the integrin αvβ3 was also reduced, while β1 integrin levels were high, with signaling to Src, Syk, and Vav compromised. The expression levels of the pattern recognition receptor Clec2 and its ligand, podoplanin, which signal to Syk and Vav, were increased, all suggesting CD82’s role in cytoskeleton assembly and its overall role for normal osteoclast function. In another study involving platelets in CD82 null mice [46], loss of CD82 resulted in reduced bleeding time in vivo. There was no difference in platelet activation, degranulation, or aggregation, but the kinetics of clot retraction was enhanced. There was increased surface expression of αIIbβ3 integrins, enhanced adhesion, and increased tyrosine kinase signaling on fibrinogen.

CD82 as a metastasis suppressor seems to associate and mediate several different proteins through regulation of a variety of signaling pathways. Since the loss of CD82 has been linked to many different cellular events, there is a high possibility that CD82 may be regulating more than one protein or one pathway in prostate cells, i.e., the presence or absence of CD82 in prostate cells may cause changes in gene expression profiles with the accompanying protein expression in these cells. Thus, studying these profiles and identifying the genes involved in these cells will help us better understand CD82’s role as a tumor metastasis suppressor in prostate cancer. Previous gene expression studies include EST sequencing [47], serial analysis of gene expression [48], differential display PCR [49], and microarray [50]. Here, we use the whole human genome gene expression microarray to identify genes regulated by CD82 in normal prostate cells that express CD82 (PrEC-31) against the same cells subject to knocked down with CD82 siRNA. Conversely, metastatic prostate cell line PC3 that does not express CD82 were compared with its clones transfected with CD82. Quantitative real-time PCR (qRT-PCR) were then used to validate the microarray results [51, 52]. Identification of differentially expressed genes in these prostate cells will provide further information on the genes CD82 may regulate to help decipher an overall role for CD82 in metastasis prevention.

## Methods

### Cell lines and cell culture

Human normal prostate epithelial cells (PrEC-31) expressing CD82 were donated by Dr. Beatrice Knudsen, University of Utah, and were cultured as previously described [53]. Briefly, cells were maintained in keratinocyte serum-free medium supplemented with human recombinant EGF and bovine pituitary extract (Gibco) and kept at 37 °C in a 5% CO_2_ incubator. Cell authentication was tested with short tandem repeat (STR) analysis. Bone-derived metastatic prostate cell line PC3 was obtained from American Type Culture Collection (ATCC). Clones of PC3: PC3-5 V (empty vector transfected; −CD82), PC3–29 and PC3–57 (transfected with pCDNA3,1(PAL)N-flag.CD82 plasmid construct to express CD82), were generated as previously described [30]. All PC3 cells were maintained in F12K medium (Invitrogen), supplemented with 10% fetal bovine serum, 2 mM glutamine, and 50 units of penicillin and 50 μg of streptomycin/ml, as previously described. When all cell cultures reached 70–80% confluence, they were trypsinized, pelleted, and stored at − 80 °C for RNA extraction. Cell lysates were also collected to perform western blot.

### siRNA silencing of PrEC-31

When PrEC-31 cells reached 70–80% confluency, they were split equally into a 6-well plate to determine the optimal CD82 siRNA concentration required for silencing CD82 expression. CD82 siRNA (5′ GAGCAGTTTCATCTCTGTC 3′) (Integrated DNA Technologies), in conjunction with siLentFect lipid reagent (Bio-Rad) was used to knock down CD82 from the PrEC-31 cells. siRNA silencing was optimized using various concentrations of siRNA (30 nM, 40 nM, 50 nM and 60 nM), with 5 μL of siLentFect lipid reagent for 48 h. according to the manufacturer’s instructions. Upon visualization of CD82 knockdown with Western blot described below, 40 nM of CD82 siRNA concentration was selected as the optimal concentration for CD82 mass silencing. Thus, PrEC-31 cells were cultured in 10 cm plates and mass silencing was performed with 40 nM siRNA with 5 μL of siLentfect lipid reagent for 48 h. PrEC-31 cells were also transfected with 40 nM scrambled siRNA as controls. After 48 h. of incubation, the cells were trypsinized, pelleted, and stored at − 80 °C for RNA extraction. CD82 knockdown in CD82 siRNA and control cells was confirmed with western blot as described below.

### Western blot

After the appropriate silencing period, the media was removed from the cells and washed with 1X PBS. Cells were lysed with MAP kinase lysis buffer [54] containing protease inhibitor cocktail. Fifty microliter of MAPK lysis buffer was added to the 6-well plates or 200 μl of MAPK lysis buffer was added to the 10 cm plates and left on ice for 30 min. The supernatant from the lysed cells was separated by centrifugation at 12,000 rpm at 4 °C for 10 min. The protein concentrations in the cell lysates were determined using Bicinchoninic Acid (Pierce Chemical Company) and subject to gel electrophoresis. Equal amounts of the protein samples were loaded onto a 10% precast Tris-Glycine gel (Novex) along with Pierce 3-Color protein molecular weight marker mix (Thermo Scientific), ran at 125 V for 90 min and the blot transferred to a Polyvinylidene Fluoride (PVDF) membrane. The blot was subject to western blot analysis using CD82 (TS82b) antibodies (Abcam). Protein bands on the blot were visualized using SupersignalWest Pico chemiluminescent reagent (Thermo Scientific) per the manufacturer’s instructions and images were captured using an UVP EpiChemi3 Darkroom UV transilluminator, attached to a Hamamatsu camera.

### Trizol precipitation and RNA isolation

The cells were trypsinized and centrifuged at 1000 rpm for 5 min before washing with 1X PBS to collect the cell pellet. Trizol precipitation and RNA isolation were then performed as previously described [55]. To remove genomic DNA contamination, a mixture of 3.5 μL DNase buffer, 2 μL RNAse inhibitor, and 2 μL DNase I was added to the RNA followed by a 20 min incubation in a heat block at 37 °C. Total RNA was isolated and purified using RNeasy Mini Kit (Qiagen) according to the manufacturer’s instructions. The RNA was then quantified using a NanoVue spectrophotometer (General Electric).

### Gene expression microarray assay

Metastatic prostate cells (PC3-5 V, PC3–29, and PC3–57), normal prostate cells PrEC-31 (+CD82), and PrEC-31 treated with scrambled siRNA (−CD82) were used to perform gene expression microarray assay. The 4 × 44 K whole human genome two-color microarray (Agilent) was used with quick amp labeling protocol. First, RNA was spiked with Spike A and Spike B mixes for cyanin-3 (cy-3, green) and cyanin-5 (cy-5, red) dyes, respectively. The RNA was then reverse transcribed to the first and second strand of cDNA. cRNA was transcribed from the second-strand of cDNA and labelled with either cy-3 or cy-5. Labeled cRNA were cleaned up using Rneasy mini spin column kit (Qiagen) before quantification with a NanoDrop. cRNA were then fragmented for 30 min with fragmentation buffer before hybridization overnight in the microarray slide at 65 °C. The microarray array slide was then washed with wash buffers 1 and 2 and was scanned using a microarray scanner (Agilent). Microarray probe featured information was extracted using Agilent Feature Extraction Software according to manufacturer’s instructions. The results were stored as raw data files in excel sheets.

### Microarray data analysis

Data Analysis was performed on three sets of microarray raw data, comparing PC3–29 (+CD82) vs. PC3-5 V (−CD82), PC3–57 (+CD82) vs. PC3-5 V (−CD82), and PrEC-31 (+CD82) vs. PrEC-31 knocked down with CD82 siRNA (−CD82), using Bioconductor R, a statistical programming environment. First, the library was loaded, and the target files were imported into the R workspace. The required fields such as gProcessedSignal, rProcessedSignal, gProcessedSigError, rProcessedSigError were read from the target files using the “read.maimages” command. A matrix was created for both red and green process signals using the “matrix” command. The data was then subjected to background correction and then normalization with loess method using the “normalizeWithinArrays” command. Normalized data was then filtered for positive and negative controls and duplicate probe values were aggregated using the “aggregate” command. Agilent probe names were then annotated with Entrez gene names and gene symbols. Commands for MA plots were then executed for a visual comparison of raw and normalized data for all three comparisons. Statistical analysis of the data was performed before which a model matrix was created where control and treatments were specified (−CD82 vs. +CD82). Linear model analysis was performed in Limma using Bayes fit method where t-test was performed between the control and treatment. The top differentially expressed genes with *P* < 0.05 were listed after adjusting with false discovery rate method. Heat maps for the top 25 genes were generated using R programming. Parametric gene set enrichment analysis (PGSEA) library was loaded and Smc (significant multivariate correlation) plots were drawn by specifying the window dimensions.

### Pathway analysis

Pathway analysis was performed for all statistical significantly expressed genes using the EASE software version 2.0 [56]. Briefly, gene symbols for each top 100 differentially expressed gene list were pasted and then to “Find Over-represented Gene category” was selected. Next, the list of all 44 K gene symbols that were present on the microarray slide was pasted and the analysis was run.

### qRT-PCR primer design and efficiency test

qRT-PCR was performed on all 5 cell lines (PrEC-31 +/−CD82, PC3-5 V, PC3–57, and PC3–29), to validate the microarray results. Two significantly differentially expressed genes: RUNX family transcription factor 3 (*RUNX3*) and trefoil factor 3 (*TFF3*) that were common among all 3 data sets and were upregulated in +CD82 cell lines (PrEC-31 + CD82, PC3–57, and PC3–29) compared to -CD82 cell lines (PrEC-31 -CD82 and PC3-5 V) and with a LogFC value of 1.4 or above required for qRT-PCR validation [57] were selected. Primers for these genes and a normalizer gene (β-actin) were designed using NCBI Primer Blast tool and Biology Workbench. All primers were designed to span at least one exon-exon junction. These primers were tested for the presence of primer dimers and hairpins using Lasergene Primer Select tool. Primer sequences that had the least number of dimers and hairpins were selected and custom made by Integrated DNA Technologies. Lyophilized forward and reverse primers were reconstituted in sterile distilled water and diluted to 5 μM for qRT-PCR assays. For primer efficiency test, 2 μg of RNA from either PC3-5 V, PC3–29, or PC3–57 was converted to cDNA using cDNA reverse transcriptase kit (Applied Biosystems) with the manufacturer’s instructions and RNase inhibitor. 150 nM final concentration of each primer (forward and reverse) with serially diluted cDNA (1:25, 1:100, 1:400) was amplified with Brilliant II SYBR green qPCR master mix kit (Stratagene) and fluorescent intensity detected in a MX3000P qPCR machine (Stratagene). For each gene, those without cDNA template were assigned as negative controls and all reactions were performed in triplicate. Reference dye was used for all efficiency tests with both SYBR and ROX dyes examined. The concentration of each sample was known to calculate the efficiency using a standard curve. All thermal profile settings were left as default except annealing temperature raised to 60 °C and the dissociation curve cycles were selected. Amplification plots, dissociation curves, and standard curves were then analyzed using threshold cycle (Ct) values and primers with the highest efficiency were used for comparative quantification of the genes (Table 1).

**Table 1:** qRT-PCR primers for validating microarray gene expression

	Primer orientation	Primer sequence (5′-3′)	Melting Temperature (Tm^0^C)	GC%
*RUNX3*	Forward primer	GACAGCCCCAACTTCCTCT	56.9	57.8
	Reverse primer	CACAGTCACCACCGTACCAT	57.0	55
*TFF3*	Forward primer	TCAAGCCCCTGCAGGAAGCAG	62.4	61.9
	Reverse primer	GCCGGGAGCAAAGGGACAGA	62.4	65
β-actin	Forward primer	AGCACTGTGTTGGCGTACAG	57.9	55
	Reverse primer	CTCTTCCAGCCTTCCTTCCT	56.4	55

### Validating microarray results with qRT-PCR

The gene expression levels of *RUNX3* and *TFF3* in all cell lines was examined. β-actin was used as the normalizer gene and 1:10 dilutions of cDNA were used. Gene expression in PC3–57 and PC3–29 cells (+CD82) was assessed with PC3-5 V cells (−CD82) as a calibrator, while the PrEC-31 knocked down cells with CD82 siRNA (−CD82) was the calibrator for the normal prostate PrEC-31 (+CD82) cells. All samples were run in triplicate and reference dye ROX was used. qRT-PCR was performed as described above. Threshold cycle values were obtained to assess the gene expression. Student t-tests were performed to compare the gene expression and *p* values less than 0.05 were regarded as statistically significant.

## Results

### CD82 siRNA silencing in normal prostate cells and restoration in metastatic cells

**Fig. 1:**
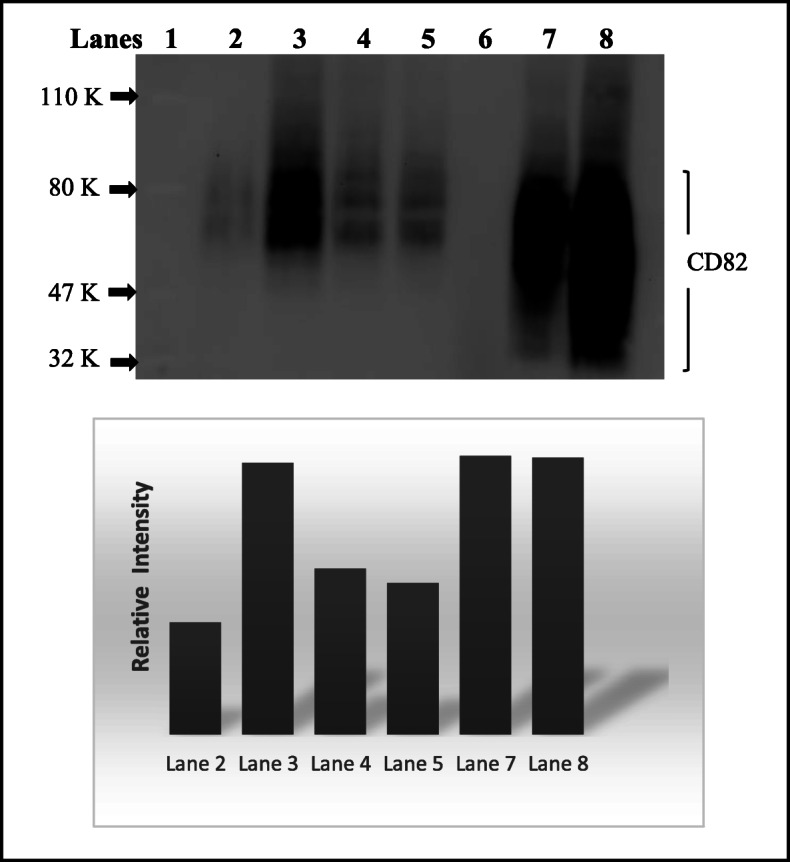
Western blot of CD82 protein expression in prostate cancer cell lines. Lane 1. Protein ladder with Phosphorylase B (110 K), BSA (80 K), Ovalbumin (47 K), and Carbonic Anhydrase (32 K). Lane 2. PC3-5 V metastatic prostate clonal cells with empty vector, Lane 3. PrEC-31 transfected with 40 nM of scrambled siRNA. Lane 4 and 5. PrEC-31 transfected with 30 nM and 40 nM of CD82 siRNA, respectively. Lane 6. empty. Lane 7 and 8. PC3–29 and PC3–57 clonal cells. Restored with CD82, respectively. A heavily glycosylated CD82 runs as a wide band between 30 and 90 KDa. Below is a graph that represents the relative intensity of the CD82 band in different lanes, based on the densitometric analysis of the blot. An uncropped full-length blot is presented in supplementary Fig. S9

We showed that CD82 can be silenced or restored in prostate cell lines (Fig. 1). Treatment with CD82 siRNA generated close to 50% knockout of CD82 expression in normal prostate cells (lanes 4 and 5) compared to treatment with scrambled siRNA (lane 3). The siRNA knockdown was close to comparison to CD82 expression in metastatic prostate PC3 cells without CD82 (clone PC3-5 V, lane 2). PC3 clones with CD82 reexpressed (PC3–29 and PC3–57, lane 7 and 8, respectively) showed restoration of CD82 with expression levels similar or higher than normal prostate cells PrEC-31 that expressed CD82 (lane 3). An uncropped gel blot is presented in supplementary Fig. S9.

### Microarray data normalization

The first preprocessing step for microarray data is the log transformation of signal intensity ratios. However, dye labeling, especially in 2-color microarray, can create nonlinear bias in the log ratios and compromise raw microarray data. Thus, microarray raw data need to be normalized before analysis. A MA plot enables visualization between intensity and difference between 2 data stores for each microarray probe. MA plots generated from raw data vs. normalized data for each 2-color microarray used in this study were compared to show successful normalization process (Supplemental Fig. S1-S3). The normalized data were then used to obtain the list of differentially expressed genes.

### Gene expression profiles of +CD82 and -CD82 prostate cells

**Fig. 2:**
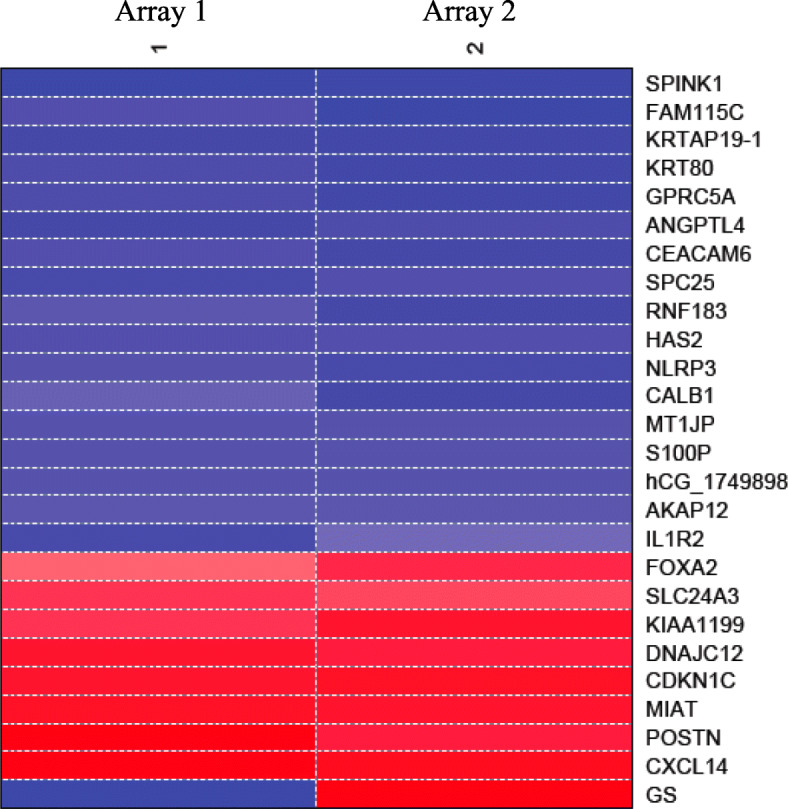
Heat map of top 25 differentially expressed genes in PrEC-31 (+/−CD82) cells. GS: graphic scale for the array, where red represents downregulation and blue represents upregulation of a gene in the normal PrEC (+CD82) compared to siRNA treatment sample PrEC (−CD82). Columns 1, 2 represent the two arrays used i.e., array 1 and array 2 as a result of dye swapping

**Fig. 3:**
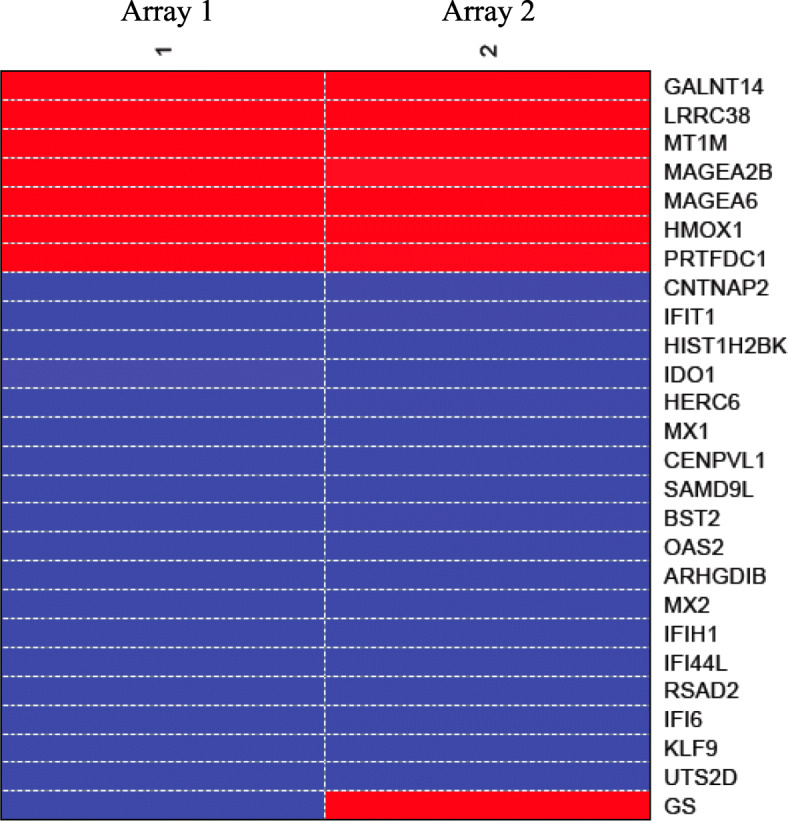
Heat map of top 25 differentially expressed genes in PC3–57 vs. PC3-5 V cells. GS: graphic scale for the array, where red represents upregulation and blue represents downregulation of a gene in the treatment PC3–57 (+CD82) compared to control PC3-5 V (−CD82). Columns 1, 2 represent the two arrays used i.e., array 1 and array 2 as a result of dye swapping

**Fig. 4:**
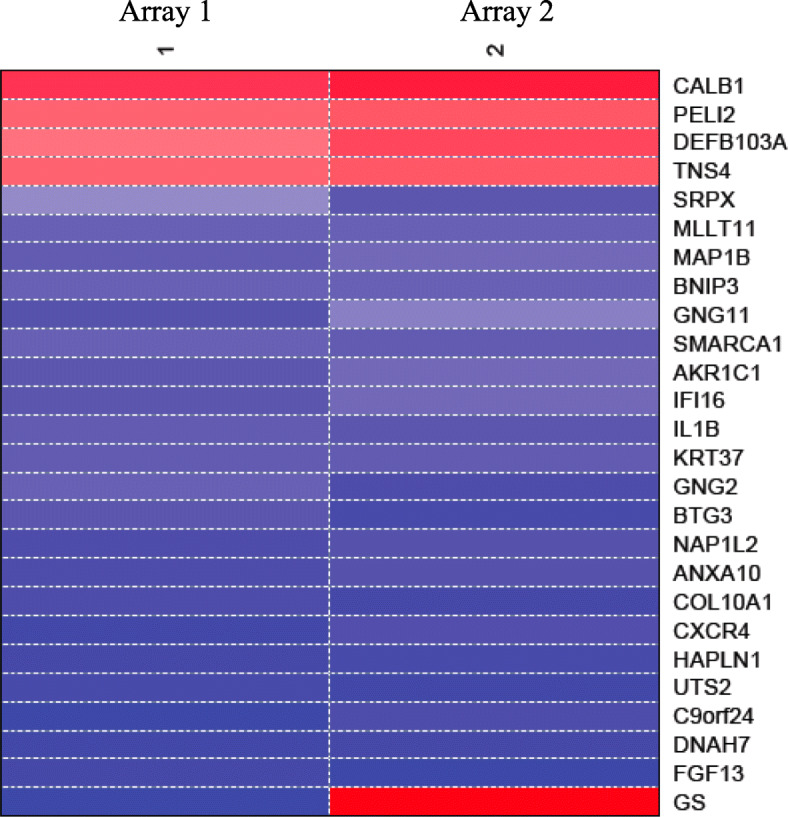
Heat map of top 25 differentially expressed genes in PC3–29 vs. PC3-5 V cells. GS: graphic scale for the array, where red represents upregulation and blue represents downregulation of a gene in the treatment PC3–29 (+CD82) compared to control PC3-5 V (−CD82). Columns 1, 2 represent the two arrays used i.e., array 1 and array 2 as a result of dye swapping

Gene expression level in cells expressing CD82 vs. those that do not express CD82 was compared with t-test and t-values were generated. Differentially expressed genes with *P* < 0.05 were identified and top 25 differentially expressed gene for PrEC-31 (+CD82) vs. PrEC-31 (−CD82), PC3–57 (+CD82) vs. PC3-5 V (−CD82), and PC3–29 (+CD82) vs. PC3-5 V (−CD82) (Table 2, 3, 4) were used to generate their respective gene expression profiles in heatmaps (Figs. 2, 3, 4).

**Table 2:** List of top 25 differentially expressed genes between PrEC (+CD82) vs. PrEC (−CD82) cells

Gene Name	Gene ID	Gene Description	logFC	t-value	*p*-value
*SPINK1*	NM_003122	*Homo sapiens* serine peptidase inhibitor, Kazal type 1	4.049	20.68	2.91E-08
*FAM115C*	NM_001130025.1	Homo sapiens family with sequence similarity 115, member C	3.492	13.18	9.91E-07
*KRTAP19–1*	NM_181607	Homo sapiens keratin associated protein 19–1	3.488	21.92	1.83E-08
*KRT80*	NM_182507	Homo sapiens keratin 80	3.372	19.55	4.53E-08
*GPRC5A*	NM_003979	Homo sapiens G protein-coupled receptor, familyC, group 5, member A	3.352	17.86	9.26E-08
*ANGPTL4*	NM_139314	Homo sapiens angiopoietin-like 4, transcript variant 1	3.243	18.89	5.96E-08
*CEACAM6*	NM_002483	Homo sapiens carcinoembryonic antigen-related cell adhesion molecule 6	3.166	17.55	1.06E-07
*SPC25*	NM_020675.3	Homo sapiens NDC80 kinetochore complex component, homolog (*S. cerevisiae*)	3.156	19.26	5.10E-08
*RNF183*	NM_145051	Homo sapiens ring finger protein 183	3.058	13.93	6.45E-07
*HAS2*	NM_005328	Homo sapiens hyaluronan synthase 2	3.009	18.95	5.81E-08
*NLRP3*	NM_001079821	Homo sapiens NLR family, pyrin domain containing 3	2.999	16.22	1.97E-07
*CALB1*	NM_004929	Homo sapiens calbindin 1, 28 kDa	2.879	10.61	5.20E-06
*MT1JP*	AF348994	Homo sapiens metallothionein 1 J (pseudogene)	2.796	17.52	1.08E-07
*S100P*	NM_005980	Homo sapiens S100 calcium binding protein P	2.786	17.39	1.14E-07
*hCG_1749898*	NM_001165252.1	Homo sapiens keratin associated protein 2–4-like	2.769	17.41	1.13E-07
*AKAP12*	NM_144497	Homo sapiens A kinase (PRKA) anchor protein (gravin) 12, transcript variant 2	2.677	16.86	1.45E-07
*IL1R2*	NM_004633	Homo sapiens interleukin 1 receptor, type II, transcript variant 1	2.657	10.31	6.47E-06
*FOXA2*	NM_021784.4	Homo sapiens forkhead box A2	− 2.521	− 13.47	8.40E-07
*SLC24A3*	NM_020689.3	Homo sapiens solute carrier family 24 (sodium/potassium/calcium exchanger), member 3	− 2.591	−15.84	2.38E-07
*KIAA1199*	NM_018689	Homo sapiens KIAA1199	−2.830	−15.95	2.25E-07
*DNAJC12*	NM_021800	Homo sapiens DNAJ (Hsp40) homolog, subfamily C, member 12, transcript variant 1	−3.081	−19.29	5.04E-08
*CDKN1C*	NM_000076	Homo sapiens cyclin-dependent kinase inhibitor 1C	−3.227	−20.16	3.56E-08
*MIAT*	NR_003491.2	Homo sapiens myocardial infarction associated transcript (non-protein coding)	−3.241	− 20.21	3.50E-08
*POSTN*	NM_006475	Homo sapiens periostin, osteoblast specific factor	−3.408	−14.07	5.98E-07
*CXCL14*	NM_004887	Homo sapiens chemokine (C-X-C motif) ligand 14	−3.774	−19.93	3.89E-08

**Table 3:** List of the top 25 differentially expressed genes between PC3–57 (+CD82) vs. PC3-5 V (−CD82) cell lines

Gene Name	Gene ID	Gene Description	logFC	t-value	*p*-value
*GALNT14*	NM_024572	Homo sapiens UDP-N-acetyl-alpha-D-galactosamine:polypeptide N-acetylgalactosaminyltransferase 14	4.836	28.08	1.18E-09
*LRRC38*	CR622769	Homo sapiens leucine rich repeat containing 38	4.452	25.60	2.56E-09
*MT1M*	NM_176870	Homo sapiens metallothionein 1 M	4.064	20.80	1.45E-08
*MAGEA2B*	NM_153488	Homo sapiens melanoma antigen family A, 2B	4.003	16.87	8.22E-08
*MAGEA6*	NM_175868	Homo sapiens melanoma antigen family A, 6, transcript variant 2	3.881	24.87	3.27E-09
*HMOX1*	NM_002133	Homo sapiens heme oxygenase (decycling) 1	3.851	23.67	4.94E-09
*PRTFDC1*	NM_020200	Homo sapiens phosphoribosyl transferase domain containing 1	3.845	21.87	9.55E-09
*UTS2D*	AK090630	Homo sapiens urotensin 2 domain containing	−5.428	−18.38	4.06E-08
*KLF9*	NM_001206	Homo sapiens Kruppel-like factor 9.	−5.01	−32.08	3.86E-10
*IFI6*	NM_022873	Homo sapiens interferon, alpha-inducible protein 6, transcript variant 3	−5.002	−20.81	1.44E-08
*RSAD2*	NM_080657	Homo sapiens radical S-adenosyl methionine domain containing 2	−4.812	−28.08	1.18E-09
*IFI44L*	NM_006820	Homo sapiens interferon-induced protein 44-like	−4.803	−30.10	6.60E-10
*IFIH1*	NM_022168	Homo sapiens interferon induced with helicase C domain 1	−4.775	−30.06	5.78E-10
*MX2*	NM_002463	Homo sapiens myxovirus (influenza virus) resistance 2 (mouse)	−4.572	−28.68	9.89E-10
*ARHGDIB*	NM_001175	Homo sapiens Rho GDP dissociation inhibitor (GDI) beta	−4.568	−25.62	2.55E-09
*OAS2*	NM_016817	Homo sapiens 2′-5′-oligoadenylate synthetase 2, transcript variant 1	−4.445	−24.84	3.30E-09
*BST2*	NM_004335	Homo sapiens bone marrow stromal cell antigen 2	−4.338	−16.78	8.61E-08
*SAMD9L*	NM_152703	Homo sapiens sterile alpha motif domain containing 9-like	−4.309	−27.31	1.49E-09
*CENPVL1*	NR_033772	Homo sapiens centromere protein V-like 1	−4.294	−23.49	5.24E-09
*MX1*	NM_002462	Homo sapiens myxovirus (influenza virus) resistance 1, interferon-inducible protein p78 (mouse)	−4.147	−24.49	3.71E-09
*HERC6*	NM_017912	Homo sapiens hect domain and RLD 6	−4.059	−21.98	9.15E-09
*IDO1*	NM_002164.4	Homo sapiens indoleamine 2,3-dioxygenase 1	− 4.029	− 14.25	3.29E-07
*HIST1H2BK*	NM_080593	Homo sapiens histone cluster 1, H2bk	−3.983	−25.10	3.02E-09
*IFIT1*	NM_001548	Homo sapiens interferon-induced protein with tetratricopeptide repeats 1	−3.914	− 20.17	1.87E-08
*CNTNAP2*	NM_014141	Homo sapiens contactin associated protein-like 2	−3.886	−19.95	2.05E-08

**Table 4:** List of top 25 differentially expressed genes between PC3–29 (+CD82) vs. PC3-5 V (−CD82) cells

Gene Name	Gene ID	Gene Description	logFC	t-value	*p*-value
*CALB1*	NM_004929	Homo sapiens calbindin 1, 28 kDa	2.759	10.18	2.56E-24
*PELI2*	NM_021255	Homo sapiens pellino homolog 2	2.246	8.28	1.22E-16
*DEFB103A*	NM_018661	Homo sapiens defensin, beta 103A	2.216	8.17	3.14E-16
*TNS4*	NM_032865	Homo sapiens tensin 4	2.212	8.16	3.48E-16
*SRPX*	NM_006307.4	Homo sapiens sushi-repeat-containing protein, X-linked	−2.198	− 8.10	5.30E-16
*FGF13*	NM_004114	Homo sapiens fibroblast growth factor 13, transcript variant 1A	−3.955	−14.58	3.68E-48
*DNAH7*	NM_018897	Homo sapiens dynein, axonemal, heavy chain 7	−3.552	−13.09	3.55E-39
*C9orf24*	NM_032596	Homo sapiens chromosome 9 open reading frame 24, transcript variant 1	−3.504	−12.91	3.63E-38
*UTS2*	NM_021995	Homo sapiens urotensin 2, transcript variant 1	−3.436	−12.66	8.93E-37
*HAPLN1*	NM_001884	Homo sapiens hyaluronan and proteoglycan link protein 1	−3.351	−12.35	4.72E-35
*CXCR4*	NM_001008540	Homo sapiens chemokine (C-X-C motif) receptor 4, transcript variant 1	−3.303	−12.17	4.14E-34
*COL10A1*	NM_000493	Homo sapiens collagen, type X, alpha 1	−3.270	−12.06	1.8E-33
*ANXA10*	NM_007193	Homo sapiens annexin A10	−3.014	−11.11	1.09E-28
*NAP1L2*	NM_021963	Homo sapiens nucleosome assembly protein 1-like 2	−2.987	−11.01	3.32E-28
*BTG3*	NM_006806	Homo sapiens BTG family, member 3	−2.941	−10.84	2.16E-27
*GNG2*	NM_053064	Homo sapiens guanine nucleotide binding protein (G protein), gamma 2	−2.689	−9.92	3.54E-23
*KRT37*	NM_003770	Homo sapiens keratin 37	−2.517	−9.28	1.70E-20
*IL1B*	NM_000576	Homo sapiens interleukin 1, beta	−2.493	−9.19	3.89E-20
*IFI16*	NM_005531	Homo sapiens interferon, gamma-inducible protein 16	−2.439	−8.99	2.42E-19
*AKR1C1*	NM_001353	Homo sapiens aldo-keto reductase family 1, member C1 (dihydrodiol dehydrogenase 1; 20-alpha (3-alpha)-hydroxysteroid dehydrogenase)	−2.418	−8.91	4.96E-19
*SMARCA1*	NM_003069	Homo sapiens SWI/SNF related, matrix associated, actin dependent regulator of chromatin, subfamily a, member 1, transcript variant 1	−2.341	−8.63	6.14E-18
*GNG11*	NM_004126	Homo sapiens guanine nucleotide binding protein (G protein), gamma 11	−2.309	−8.51	1.69E-17
*BNIP3*	NM_004052	Homo sapiens BCL2/adenovirus E1B 19 kDa interacting protein 3	−2.296	−8.46	2.59E-17
*MAP 1B*	NM_005909	Homo sapiens microtubule-associated protein 1B, transcript variant 1	−2.293	−8.45	2.82E-17
*MLLT11*	NM_006818	Homo sapiens myeloid/lymphoid or mixed lineage leukemia (trithorax homolog, Drosophila); translocated to, 11	−2.290	−8.44	3.07E-17

Top 25 gene lists did not show overlap genes between the three arrays except calbindin 1 (*CALB1*) which was significantly upregulated (2.8 log-fold change or logFC) in PrEC-31 (+CD82) and PC3–29 (+CD82) cells. In PrEC-31 (+CD82) vs. PrEC-31 (−CD82) cells, the most up- and down-regulated genes were serine peptidase inhibitor kazal type 1 (*SPINK1*; 4 logFC) and C-X-C motif chemokine ligand 14 (*CXCL14*; − 3.8 logFC) respectively. The most upregulated gene was polypeptide N-acetylgalactosaminyltransferase 14 (*GALNT14*; 4.8 logFC) in PC3–57 and calbindin 1 (*CALB1*; 2.8 logFC) in PC3–29 while the most down-regulated gene was urotensin 2 (*UTS2D*; − 5.4 logFC) in PC3–57 and fibroblast growth factor 13 (*FGF13*; − 4 logFC) in PC3–29, when compared with PC3-5 V cells.

### Pathway analysis

Pathway analysis was performed to further explain the complex mechanisms of CD82 in the prostate cell lines. Top 100 significantly differentially expressed genes that were mutually inclusive in all 3 data sets—PC3–29 (+CD82), vs. PC3-5 V (−CD82), PC3–57 (+CD82) vs. PC3-5 V (−CD82), and PrEC-31 (+CD82) vs. PrEC-31 knocked down with CD82 siRNA (−CD82) were analyzed. The data showed that pathways related with cell proliferation and angiogenesis, migration and invasion, cell death, cell cycle, signal transduction, and metabolism were highly enriched (Table 5).

**Table 5:** Key pathways regulated by CD82 in prostate cancer cells from top 100 significant genes from all three data array sets

Gene Name	Gene ID	Gene Description	PC3–57 Array (logFC)	PC3–29 Array (logFC)	PrEC-31 Array (logFC)
**Cell proliferation and angiogenesis**					
*ANXA3*	NM_002754.3	Homo sapiens annexin A3	+ 0.504	+ 1.302	+ 1.218
*MAPK3*	NM_002754	Homo sapiens mitogen-activated protein kinase 13	+ 0.715	+ 0.874	+ 0.816
*LXN*	NM_020169	Homo sapiens latexin	+ 2.255	+ 1.220	+ 1.323
*RUNX3*	NM_001031680	Homo sapiens runt-related transcription factor 3, transcript variant 1	+ 2.539	+ 1.446	−1.140
*PIK3CA*	NM_006218.2	Homo sapiens phosphoinositide-3-kinase, catalytic, alpha polypeptide	−0.648	− 0.903	+ 0.523
*CDKN1C*	NM_000076	Homo sapiens cyclin-dependent kinase inhibitor 1C (p57, Kip2)	−1.105	− 1.319	− 3.227
*BST2*	NM_004335	Homo sapiens bone marrow stromal cell antigen 2	−4.338	−1.592	+ 0.926
**Migration & Invasion**					
*TFF3*	NM_003226	Homo sapiens trefoil factor 3 (intestinal)	+ 3.835	+ 2.071	+ 0.926
*CXCL2*	NM_002089	Homo sapiens chemokine (C-X-C motif) ligand 2	−1.521	−1.422	+ 1.699
**Cell Apoptosis**					
*LCK*	NM_005356	Homo sapiens lymphocyte-specific protein tyrosine kinase, transcript variant 2	+ 1.377	+ 0.930	−0.777
*STEAP1*	NM_012449	Homo sapiens six transmembrane epithelial antigen of the prostate 1	−1.498	−1.912	**–**
**Cell cycle**					
*RUNX3*	NM_001031680	Homo sapiens runt-related transcription factor 3, transcript variant 1	+ 2.539	+ 1.446	−1.140
*CDKN1C*	NM_000076	Homo sapiens cyclin-dependent kinase inhibitor 1C (p57, Kip2)	−1.105	−1.319	−3.227
*LCK*	NM_005356	Homo sapiens lymphocyte-specific protein tyrosine kinase, transcript variant 2	+ 1.377	+ 0.930	−0.776
*FGF13*	NM_004114.3	Homo sapiens fibroblast growth factor 13	− 0.765	− 3.955	**–**
**Signal transduction pathways**					
*MAPK13*	NM_002754	Homo sapiens mitogen-activated protein kinase 13	+ 0.715	+ 0.874	+ 0.816
*LCK*	NM_005356	Homo sapiens lymphocyte-specific protein tyrosine kinase, transcript variant 2	+ 1.377	+ 0.930	−0.777
*PIK3CA*	NM_006218.2	Homo sapiens phosphoinositide-3-kinase, catalytic, alpha polypeptide	−0.648	− 0.903	+ 0.523
*CXCL2*	NM_002089	Homo sapiens chemokine (C-X-C motif) ligand 2	−1.521	−1.422	+ 1.699
*FGF13*	NM_004114.3	Homo sapiens fibroblast growth factor 13	− 0.765	− 3.955	**–**
*WNT5A*	NM_003392.3	Homo sapiens wingless-type MMTV integration site family, member 5A	− 0.577	− 1.094	+ 1.634
*PPFIA4*	NM_015053	Homo sapiens protein tyrosine phosphatase, receptor type, f polypeptide (PTPRF), interacting protein (liprin), alpha 4	−1.192	− 1.649	+ 2.211
**Metabolic pathways**					
*TFF3*	NM_003226	Homo sapiens trefoil factor 3 (intestinal)	+ 3.835	+ 2.071	+ 0.926
*MMP23B*	NM_006983	Homo sapiens matrix metallopeptidase 23B	+ 0.987	+ 0.889	**–**

### qRT-PCR primer efficiency

Dissociation curve, amplification plot, and standard curve of qRT-PCR were analyzed for primer efficiency of *RUNX3* and *TFF3* (Supplemental Fig. S4-S8). Dissociation curve analysis showed accurate amplification of each qRT-PCR target (Fig. S4), while amplification plot analysis showed appropriate fluorescence intensity range and exponential amplification (Fig. S5 and S7). Standard curve generated from Ct values in the amplification plot of known target quantity showed primers efficient between 86 to 99.9% (R2 = 0.998) (Fig. S6 and S8). These primers were used for comparative quantification of *RUNX3* and *TFF3* genes with qRT-PCR.

### qRT-PCR validation of microarray gene expression

**Fig. 5:**
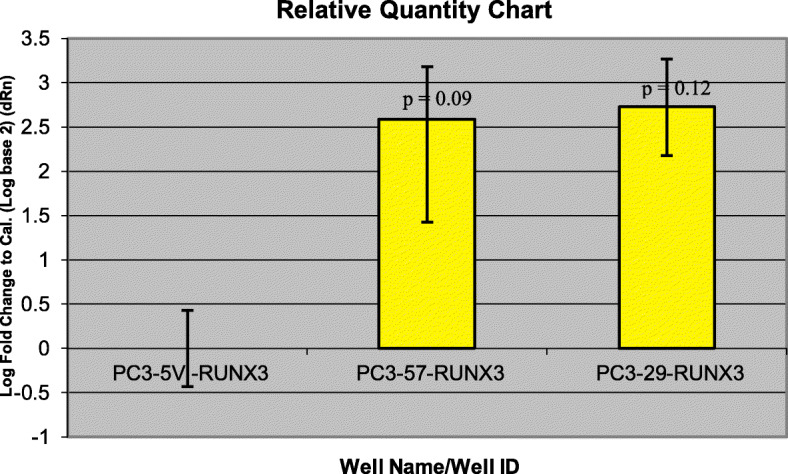
Comparative quantification for *RUNX3* gene in PC3 cell lines using qRT-PCR. PC3-5 V cell line was used as the calibrator. Yellow bars represent the log-fold change for the PC3–57 and PC3–29 cell lines compared to PC3-5 V. Fold change were initially calculated for all the three cell lines by subtracting *RUNX3* Ct values from the respective cell lines beta actin Ct values. The fold change for PC3-5 V cell lines was equaled to 0 and the values for PC3–57 and PC3–29 were calculated by comparing to PC3-5 V. A t-test performed on the final fold change values yielded *p* values of 0.12 (PC3–29) and 0.09 (PC3–57) respectively

**Fig. 6:**
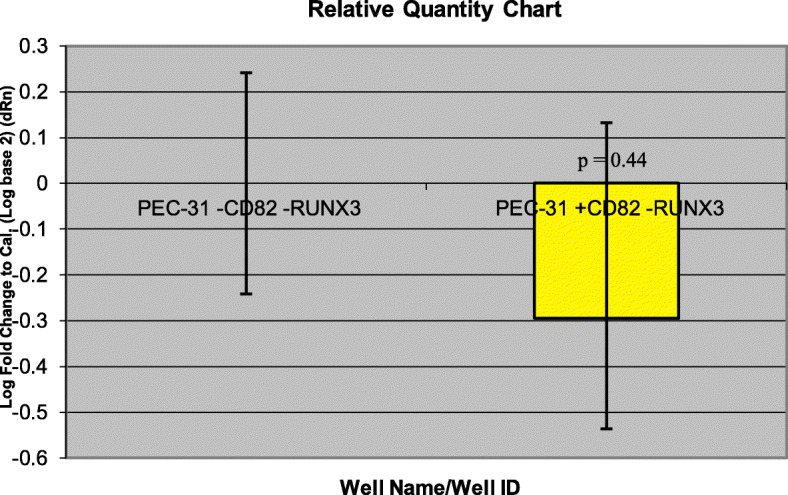
Comparative quantification for *RUNX3* gene in PrEC-31(+/− CD82) cells with qRT-PCR. PrEC-31-CD82 cell line was used as the calibrator. Yellow bars represent the log-fold change for the PrEC-31 + CD82 cell lines compared to PrEC-31-CD82. Fold change was initially calculated for both cell lines by subtracting *RUNX3* Ct values from the respective cell lines beta actin Ct values. The fold change for PrEC-31-CD82 cell line was equaled to 0 and the values for PrEC-31 + CD82 were calculated by comparing to PrEC-31-CD82. A t-test performed on the final fold change value yielded a p value of 0.44

**Fig. 7:**
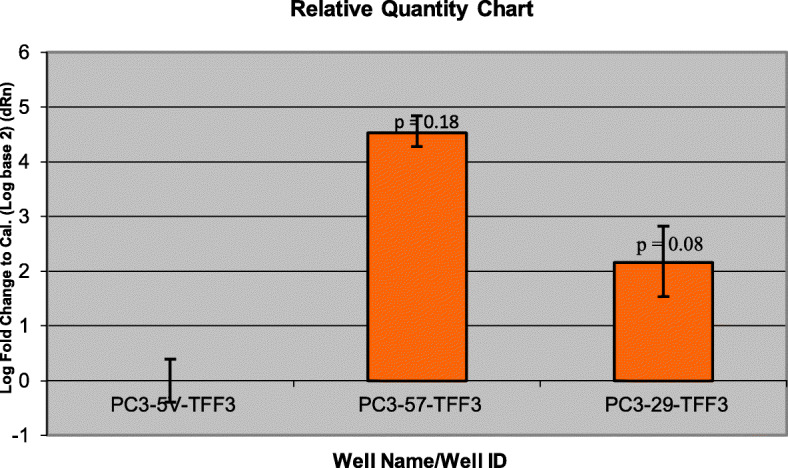
Comparative quantification for *TFF3* gene in PC3 cells using qRT-PCR. PC3-5 V cell line was used as the calibrator. Yellow bars represent the log-fold change for the PC3–57 and PC3–29 cell lines compared to PC3-5 V. Fold change was initially calculated for all the three cell lines by subtracting *TFF3* Ct values from the respective cell lines beta actin Ct values. The fold change for PC3-5 V cell line was equaled to 0 and the values for PC3–57 and PC3–29 were calculated by comparing to PC3-5 V. A t-test performed on the final fold change values yielded p values of 0.08 (PC3–29) and 0.18 (PC3–57) respectively

**Fig. 8:**
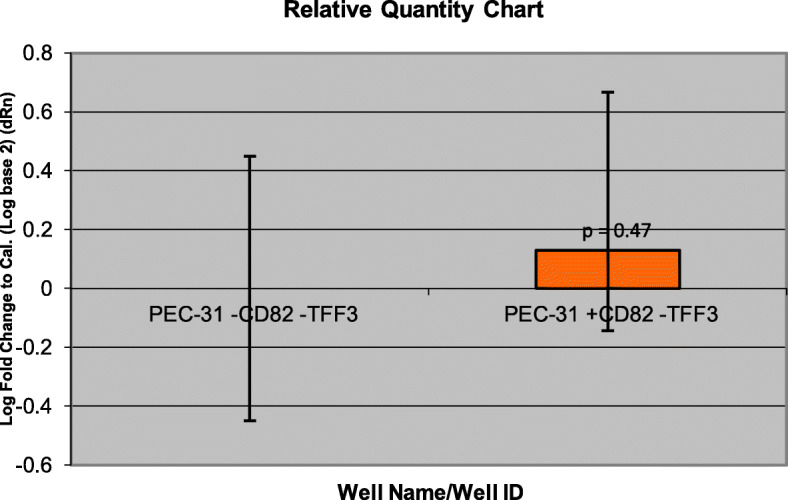
Comparative quantification for TFF3 gene in PrEC-31(+/− CD82) cells using qRT-PCR. PEC-31-CD82 cell line was used as a calibrator. Yellow bars represent the log-fold change for the PEC-31 + CD82 cell lines compared to PEC-31-CD82. Fold change was initially calculated for both cell lines by subtracting TFF3 Ct values from the respective cell lines beta actin Ct values. The fold change for PEC-31-CD82 cell line was equaled to 0 and the values for PEC-31 + CD82 were calculated by comparing to PEC-31-CD82. A t-test performed on the final fold change yielded a p value of 0.47

Gene expression data obtained from microarrays could be validated with qRT-PCR. *RUNX3* has higher gene expression in CD82-restored PC3 clones (PC3–29 and PC3–57) (Fig. 5) compared with PC3-5 V -CD82 but was downregulated in normal prostate cells by PrEC-31 + CD82 (Fig. 6), as observed from the microarray data. Student t-test comparing the final fold change difference of RUNX3 between cell lines yielded *p* values of 0.12 (PC3-5 V vs PC3–29), 0.09 (PC3-5 V vs PC3–57) and 0.44 (PrEC-31; + vs – CD82) respectively. On the other hand, *TFF3* showed higher gene expression in all +CD82 cells when compared with -CD82 cells (Figs. 7 and 8), correlated with the respective microarray data. The p values for TFF3 gene expression were 0.08 (PC3-5 V vs PC3–29), 0.18 (PC3-5 V vs PC3–57) and 0.47 (PrEC-31; + vs – CD82) respectively.

## Discussion

CD82 as tumor metastasis suppressor plays an important role in preventing primary tumor progression to a metastatic stage. Thus, CD82 is found downregulated in many metastatic human cancers. In metastatic prostate cancer cells, although c-Met signalling pathway has been identified to be regulated by CD82 (30), additional pathways may be involved. One way to further determine the genes and the pathways associated with CD82 is by identifying the differentially expressed genes in prostate cells with or without CD82. In this study, we used a whole human genome gene expression microarray to identify genes and subsequent pathways that are regulated by CD82 in normal and metastatic prostate cells (PrEC-31 and PC3).

Top 25 differentially expressed genes from the 3 microarray data sets: PrEC-31 (+CD82) vs. PrEC-31 (−CD82), PC3–57 (+CD82) vs. PC3-5 V (−CD82), and PC3–29 (+CD82) vs. PC3-5 V (−CD82) did not show overlapped results, except *CALB1* which was up-regulated in both PrEC-31 and PC3–29 CD82 expressed cells. *CALB1* encodes calcium-binding protein calbindin 1 that is thought to play a role in apoptosis inhibition. However, *CALB1* expression is reported to correlate with improved survival of patients with lung cancer [58], contradicting with reports that suggested an association of *CALB1* upregulation with cancer stemness in meningiomas [59] and senescence inhibition in ovarian cancer [60]. The discrepancy may be due to different cell types and detection techniques that were used. Our study showed downregulation of *CALB1* in CD82 negatively expressed normal and metastatic prostate cells (PrEC-31 and PC3). Low CD82 expression has been reported to correlate with increased invasiveness and decreased calcium-related cell-cell adhesion and adhesion to fibronectin in bladder cancer cell lines [61]. Although the relation between *CALB1* and CD82 is currently unknown, CD82 may interact with *CALB1* to disrupt calcium homeostasis. Low CD82 coupled with low *CALB1* may cause insufficient calcium binding to calcium ions in normal cells and osteoclasts during metastasis. This may enhance tumor development and bone metastasis in prostate cancer (as PC3 cells are from bone metastasis), as observed in metastatic breast cancer [62].

In PrEC-31 (+CD82) vs. PrEC-31 (−CD82) cells, we showed *SPINK1* as the most differentially upregulated gene. In prostate cancer, EGFR signaling pathway induces *SPINK1* trypsin inhibitor to promote EMT [63] and overexpression of *SPINK1* represents its aggressive form [64]. Although studies have shown positive association of *SPINK1* expression with biochemical recurrence and castration-resistant prostate cancer [65], there was no significant difference in *SPINK1* expression between incidental and metastatic cases [66]. The positive correlation between *SPINK1* and CD82 expression in PrEC-31 normal prostate cells remains unexplained without further studies. *CXCL14* was the most downregulated gene in PrEC-31 (+CD82) compared with PrEC-31 (−CD82). *CXCL14* expression is known to upregulate in prostate cancer and positively correlate with its tumor progression [67, 68]. *CXCL14*, as a fibroblast autocrine growth factor can act as a prostate cancer stimulator [69] and high *CXCL14* gene expression in -CD82 cells may indicate a possible link between *CXCL14* and CD82 in the tumorigenesis of prostate cancer.

In one of the two PC3 metastatic prostate clonal cells (PC3–57) restored with CD82, we found *GALNT14* as the most differentially upregulated gene when compared with CD82 negative cells. Aberrant glycosylation is a hallmark in various cancers and *GALNT14*, as a glycosyltransferase in the Golgi membrane, has been shown to promote lung-specific breast cancer metastasis by suppressing the bone morphogenetic protein signalling and activating the fibroblast growth factors to recruit macrophages for its metastatic microenvironment [70]. Here, we showed upregulation of *GALNT14* in CD82 restored metastatic prostate cells and it is uncertain whether adding CD82 caused methylation that increased *GALNT14* expression in those cells, as seen in many cancers [71] or is due to some other mechanism. *UTS2D* and *FGF13* were the most downregulated genes in PC3–57 (+CD82) and PC3–29 (+CD82) cells, respectively. *UTS2D* codes for urotensin 2 (*UTS2*), a vasoconstrictor that binds to urotensin 2 receptor (*UTS2R*) in the G protein coupled receptor (GPCR) pathway. In prostate cancer, two studies showed an association of lower *UTS2R* expression with higher Gleason score and a more advanced cancer stage [72, 73], while a recent study demonstrated the opposite results i.e., a higher *UTS2R* expression correlated with higher grade and cancer stage [74]. In PC3 cells that have lower CD82 expression (PC3-5 V -CD82), although we did not find significant changes in *UTS2R*, we detected significantly higher *UTS2D* gene expression when compared with the +CD82 cells. If CD82 is involved in *UTS2D/UTS2R* mediation, developing *UTS2R* blockers may be a potential treatment avenue for prostate cancer, as suggested by Zappavigna et al. [75]. Fibroblast growth factors (FGFs) are proteins that are involved in various important biological processes, including cell differentiation and migration. In prostate cancer, *FGF13* could act as an onco-switch [76] and its expression was higher in malignant as well as locally invasive and metastatic cells when compared with benign control cells [77]. High expression of *FGF13* regulated by E2F1 transcription factor was reported to correlate with a shorter cell migration time to metastatic sites in breast cancer [78]. Additionally, up-regulated *FGF13* gene expression was identified in highly metastatic breast cancer cells [79]. High expression of *FGF13* in metastatic prostate -CD82 cells observed in our study indicates a potential interaction between *FGF13* and CD82 to promote metastasis in prostate cancer.

A possible explanation for the discrepant top 25 differentially expressed gene lists within the 3 data sets is that different type of prostate cells were used in this study. PrEC-31 is a normal prostate cell line isolated from a patient after prostatectomy, while PC3 is a metastatic prostate cell line isolated from bone; both cell lines were also cultured in different culture conditions. Additionally, PC3-5 V, PC3–57, and PC3–29 are clonal cell lines derived from PC3. Discrepant results between PC3–57 + CD82 and PC3–29 + CD82 clonal cells when compared with PC3-5 V -CD82 cells may be due to the fact that prostate tumor can harbor multiple genetically distinct cancer clones with heterogenous *ERG+, ETS+, SPI+* and triple negative subtypes [80, 81] that can affect different gene regulation and pathways. Moreover, high vs. low metastatic cells have been known to be derived from parental PC3 cells [82]. In addition, as RNA translation to proteins is regulated by many molecular aspects, higher or lower gene expression that we identified in our microarrays may not directly reflect their protein expression. However, pathway analysis using gene expression data may provide mechanistic insight into how CD82 functions in prostate cells. We selected top 100 differentially expressed genes that were mutually inclusive in all 3 data sets to perform pathway analysis. Key pathways related with cell proliferation and angiogenesis, migration and invasion, cell death, cell cycle, signal transduction, and metabolism were identified in prostate cells, regulated by CD82. In general, these pathways are associated with oncogenesis and metastasis.

We performed qRT-PCR on *RUNX3* and *TFF3*, two genes that were consistently up regulated in all +CD82 cells (except *RUNX3* which was downregulated in PrEC-31 + CD82). Quantitative comparison of *RUNX3* and *TFF3* expression levels measured with qRT-PCR showed higher gene expression in all +CD82 cells except PrEC-31 + CD82, which was consistent with our microarray results (Table 5). A t-test comparing the log fold changes in the expression of these genes did not show statistical significance.

*RUNX3*, a runt-related transcription factor, is identified as a tumor suppressor gene in a wide variety of invasive and preinvasive epithelial and mesenchymal tumors [83]. *RUNX3* is suggested to play a significant role in promoting apoptosis and inhibition of angiogenesis, EMT, cell migration, and invasion [84]. Its tumor suppressive activity was first identified in gastric epithelial cells of *RUNX3* knockdown mice, where absence of *RUNX3* resulted in increased proliferative activity, suppressed apoptosis and decreased sensitivity to transforming growth factor beta (TGF-β) [85]. *RUNX3* has since been identified as a downstream regulator of TGF-β signaling pathway, by inducing *CDKN1A* (p21) gene expression in gastric cells [86] and upregulating the expression of proapoptotic gene *BCL2L11* (Bim) in TGF-β treated cells [87]. As a mediator of TGF-β signaling, *RUNX3* has been shown to inhibit EMT that promotes metastasis in gastric cancer [88] and hepatocellular carcinoma cells [89]. Additionally, re-expression of *RUNX3* in gastric cells in a mouse model inhibited peritoneal metastasis [90] and *RUNX3* restoration in human gastric cancer cells suppressed vascular endothelial growth factor A (VEGF A) expression, leading to inhibition of angiogenesis, growth, and metastasis [91]. In prostate cancer, reduced levels of *RUNX3* have been correlated with tumor stage and grade [92]. RUNX3 overexpression in prostate cancer cells showed inhibition in cell migration and invasion with an upregulation of tissue inhibitor of matrix metalloproteinase-2 (TIMP-2). The functional role exhibited by *RUNX3* is very similar to the role CD82 plays in prostate cells, i.e., CD82 reexpression has been shown to inhibit EMT (36), inhibit migration and invasion (30) and its down-regulation has been correlated with poor prognosis (2–4) in prostate cancer. Although we are unclear of the association between CD82 and *RUNX3* in our study, CD82 reexpression seems to promote upregulation of *RUNX3* in metastatic prostate cells.

On the other hand, *TFF3* or trefoil factor 3 is a secretory protein that plays an important part in mucosal protection by promoting cell migration and preventing apoptosis [93]. *TFF3* is secreted in various tissues, including pancreas, salivary glands, lacrimal glands, prostate, breast, uterus, respiratory tract, and hypothalamus [94, 95]. In prostate cancer, *TFF3* expression was found to be up-regulated when compared to normal prostate tissue and *TFF3* overexpression in PC3 cells was shown to increase proliferation, cell survival, and oncogenicity while reduce ionizing radiation sensitivity in prostate cells [96]. Our results showed increased *TFF3* expression levels in all +CD82 cells and it correlated with the qRT-PCR data. .

## Conclusion

In summary, even though earlier studies have explored the role of CD82 in prostate cancer and other cancer metastasis, our study was the first where we used microarray analysis to observe differential gene expression in prostate cells with and without CD82. We have identified multiple gene targets that could further be explored, including their association with CD82 in regulating prostate cancer metastasis. The significantly upregulated genes in -CD82 cells such as *CXCL14* and *FGF13* could potentially serve as biomarkers or therapeutic targets for diagnosis and treatment of prostate cancer.

## Supplementary Information

**Additional file 1.**

**Additional file 2.**

## Data Availability

All data generated or analysed during this study are included in this published article [and its supplementary information files].

## Abbreviations

PrEC-31 cells: Human normal prostate cells derived from prostectomy (express CD82)
PC3 cells: Human prostate cells derived from bone metastasis (do not express CD82)
PC3-5 V: Vector transfected PC3 cells (do not express CD82)
PC3–29 & PC3–57: Stable clones of PC3 transfected with CD82 (express CD82)
*KAI1*: Kangai 1 gene
*CALB1*: The calcium binding protein calbindin 1
*SPINK1*: Serine peptidase inhibitor kazal type 1
*GALNT14*: Polypeptide N-acetyl galactosaminyl transferase 14
*CXCL14*: C-X-C motif chemokine ligand 14
*UTS2D*: Urotensin 2
*FGF13*: Fibroblast growth factor 13
*RUNX3*: Runt-related transcription factor 3
*TFF3*: Trefoil factor 3
EMT: Epithelial to mesenchymal transition
