# Supplementary material for: Gene expression analysis of human prostate cell lines with and without tumor metastasis suppressor CD82
Source: BMC Cancer. 2020 Dec 9;20:1211. doi: 10.1186/s12885-020-07675-7 (PMC7724878; doi:10.1186/s12885-020-07675-7)
Supplement: Supplementary file 1 — Additional file 1. [file 12885_2020_7675_MOESM1_ESM.pdf]

Supplementary Figures

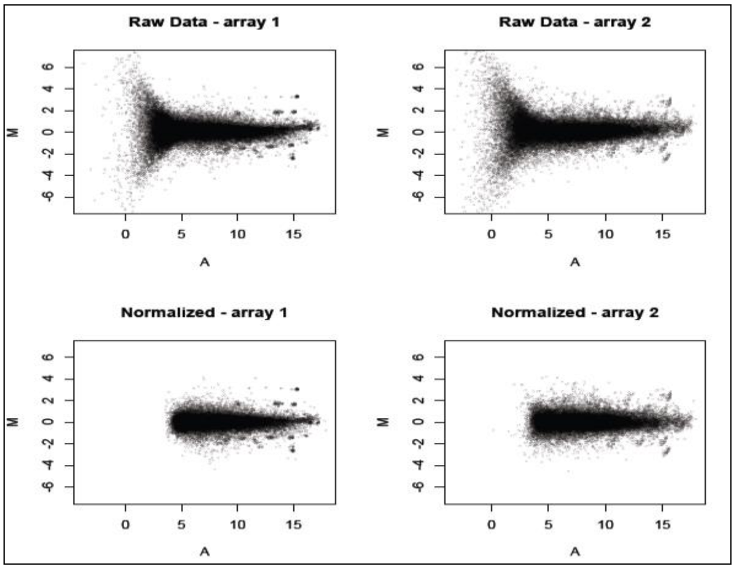

Figure S1: MA plots for PrEC-31 (+/-CD82) cell lines microarray data. Raw Data–array 1: PrEC-31 (-CD82) cell line array plot before normalization. Raw Data–array 2: PrEC-31 (+CD82) cell line array plot before normalization. Normalized Data–array 1: PrEC-31 (-CD82) cell line array plot after normalization. Normalized Data–array 2: PrEC-31 (+CD82) cell line array plot after normalization.

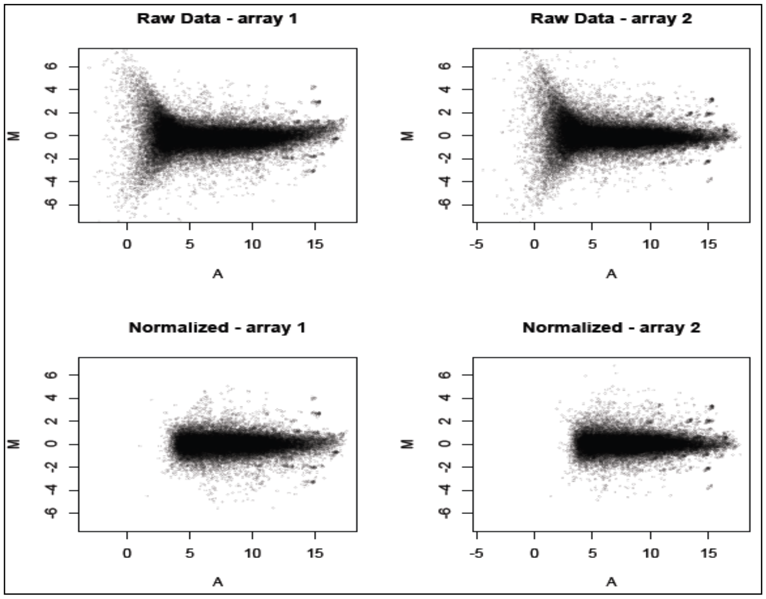

Figure S2: MA plots for PC3-5V and 57 (+/-CD82) cell lines microarray data. Raw Data–array 1: PC3-5V (-CD82) cell line array plot before normalization. Raw Data–array 2: PC3-57 (+CD82) cell line array plot before normalization. Normalized Data–array 1: PC3-5V (-CD82) cell line array plot after normalization. Normalized Data–array 2: PC3-57 (+CD82) cell line array plot after normalization.

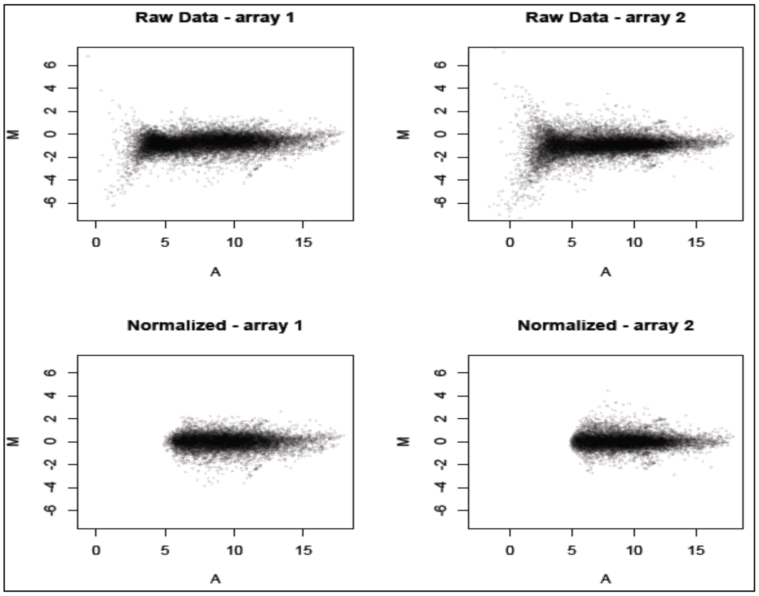

Figure S3: MA plots for PC3-5V and 29 (+/-CD82) cell lines microarray data. Raw Data–array 1: PC3-5V (-CD82) cell line array plot before normalization. Raw Data–array 2: PC3-29 (+CD82) cell line array plot before normalization. Normalized Data–array 1: PC3-5V (-CD82) cell line array plot after normalization. Normalized Data–array 2: PC3-29 (+CD82) cell line array plot after normalization.

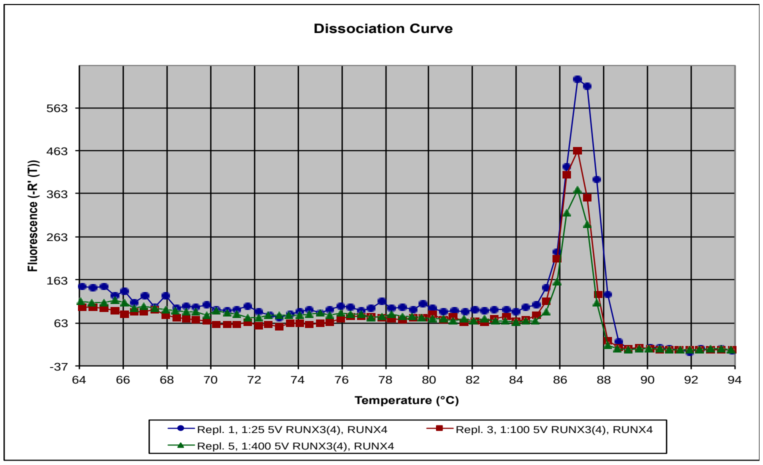

Figure S4: Dissociation curve for *RUNX3* gene primers. Primer efficiency test for *RUNX3* gene primers was performed using different dilutions of PC3-5V cDNA. The graph was plotted with fluorescence on Y-axis and temperature on X-axis. The peak represents the temperature at which the two strands of DNA were dissociated. The legend at the bottom of the graph shows what each curve represents: 1:25, 1:100, and 1:400, dilutions of PC3-5V cDNA.

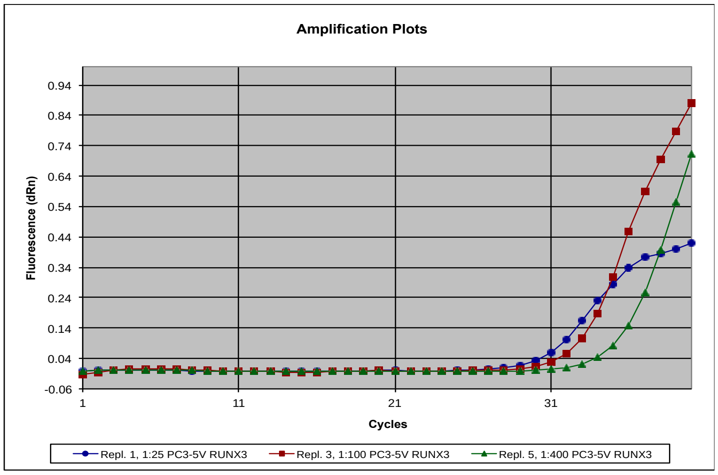

Figure S5: Amplification plot for *RUNX3* gene primers. Primer efficiency test for *RUNX3* gene primers was performed using different dilutions of PC3-5V cDNA. The graph was plotted with fluorescence on Y-axis and PCR cycle number on X-axis. The legend at the bottom of the graph shows what each curve represents: 1:25, 1:100, and 1:400, dilution of the PC3-5V cDNA. Ct values for 1:25, 1:100 and 1:400 dilution curves were 27.01, 28.85 and 30.96 respectively.

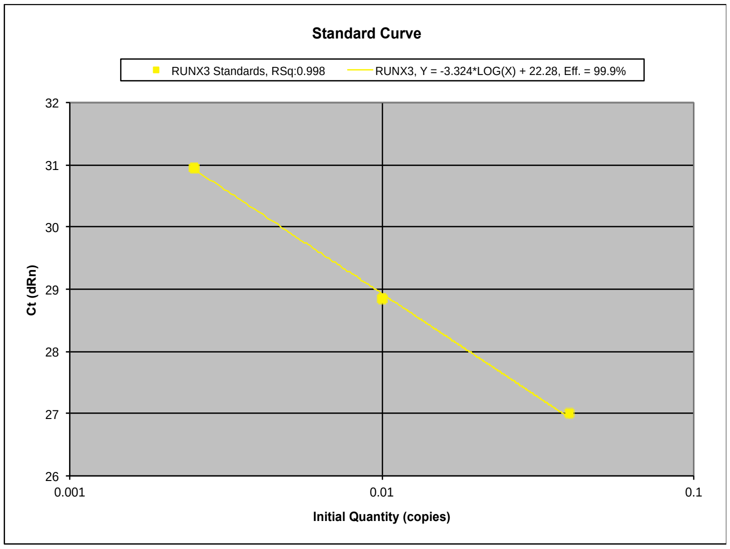

Figure S6: Standard curve for *RUNX3* gene primers. Primer efficiency for *RUNX3* gene primers was performed using different dilutions of PC3-5V cDNA. The graph was plotted with initial copy numbers (relative to cDNA dilutions) on X-axis and PCR cycle number (Figure 12) on Y-axis. Rsq and efficiency values were calculated by the program using the formula 10 (-1/slope) =Efficiency.

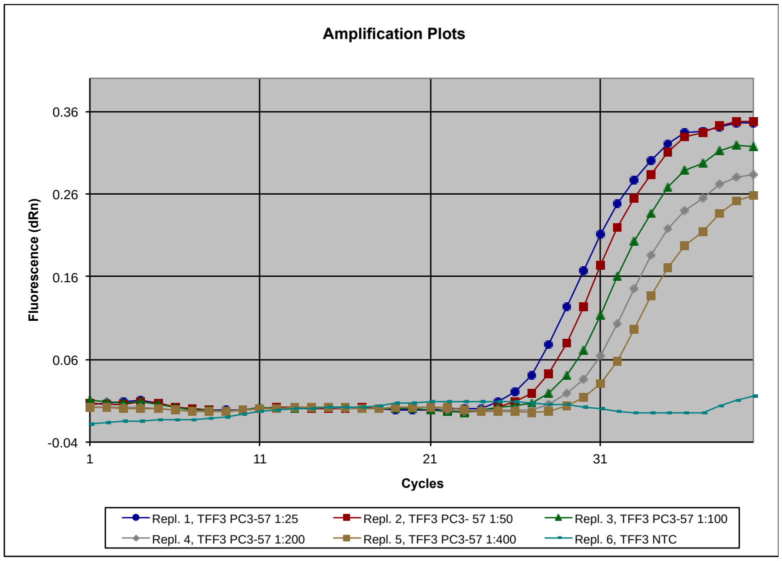

Figure S7: Amplification plot for *TFF3* gene primers. Primer efficiency test for *TFF3* gene primers was performed using different dilutions of PC3-57 cDNA. The graph was plotted with fluorescence on Y-axis and PCR cycle number on X-axis. The legend at the bottom of the graph shows what each curve represents: 1:25, 1:50, 1:100, 1:200, and 1:400, dilution of the PC3-57 cDNA. Ct values for 1:25, 1:50, 1:100, 1:200 and 1:400 dilution curves were 25.66533, 26.69377, 27.83668, 28.85065 and 30.19622 respectively.

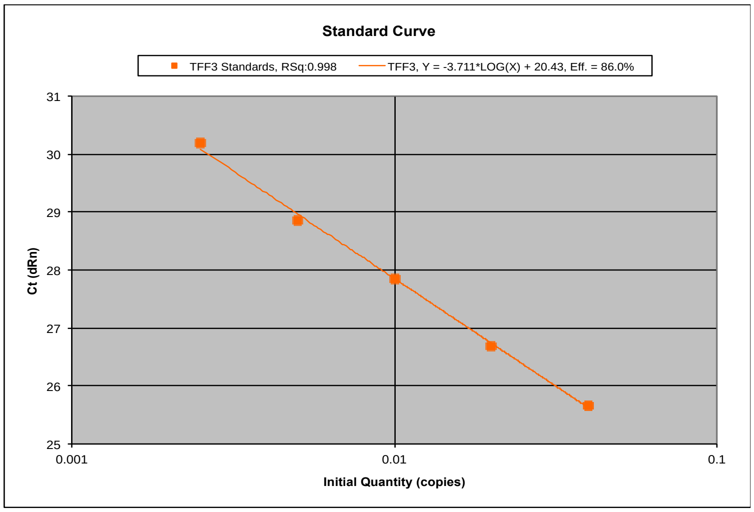

Figure S8: Standard curve for *TFF3* gene primers. Primer efficiency test for *TFF3* was performed using different dilutions of PC3-57 cDNA. The graph was plotted with initial copy numbers (relative to cDNA dilutions) on X-axis and PCR cycle number (Figure 14) on Y-axis. Rsq and efficiency values were calculated by the program using the formula 10(-1/slope) =Efficiency.

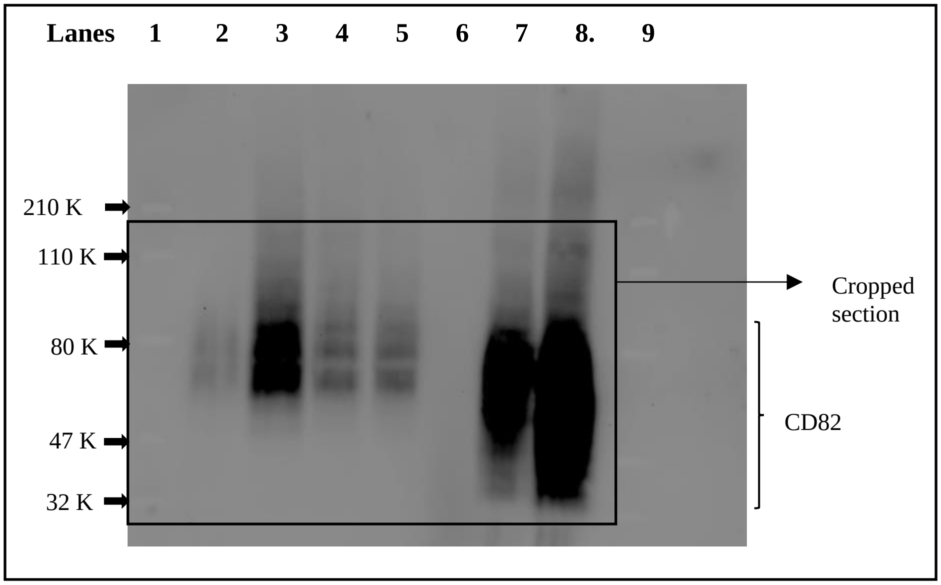

Figure S9: Western blot of CD82 protein expression in prostate cancer cell lines- the uncropped gel of figure 1. Lane 1 and 9. Protein ladder with Myosin (210K) Phosphorylase B (110K), BSA (80K), Ovalbumin (47K), and Carbonic Anhydrase (32K). Lane 2. PC3-5V metastatic prostate clonal cells with empty vector, Lane 3. PrEC-31 transfected with 40 nM of scrambled siRNA. Lane 4 and 5. PrEC-31 transfected with 30 nM and 40 nM of CD82 siRNA, respectively. Lane 6. empty. Lane 7 and 8. PC3-29 and PC3-57 clonal cells restored with CD82, respectively.
