# Supplementary material for: Gene expression analysis of human prostate cell lines with and without tumor metastasis suppressor CD82
Source: BMC Cancer. 2020 Dec 9;20:1211. doi: 10.1186/s12885-020-07675-7 (PMC7724878; doi:10.1186/s12885-020-07675-7)
Supplement: Supplementary file 2 — Additional file 2. [file 12885_2020_7675_MOESM2_ESM.pdf]

Supplementary Figure S9: Uncropped blot

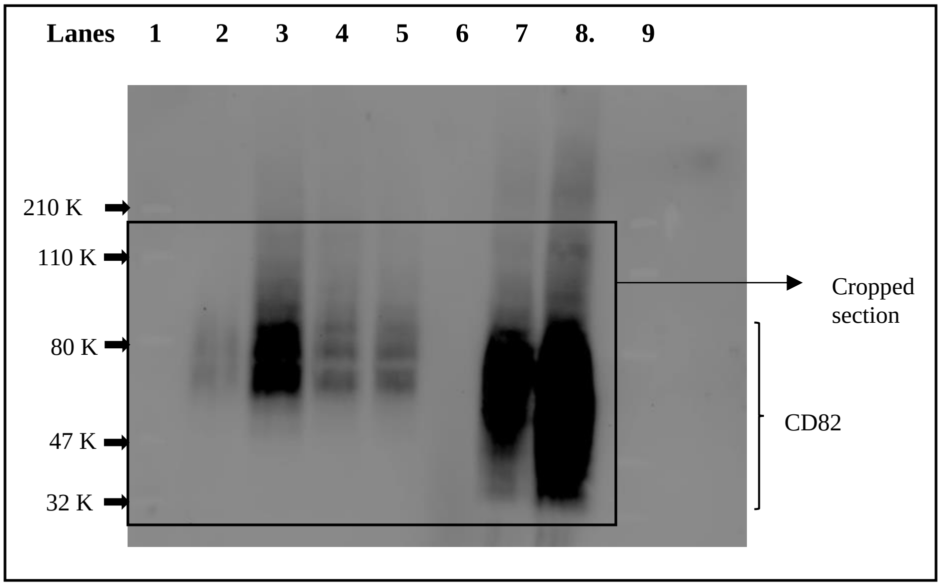

Figure S9: Western blot of CD82 protein expression in prostate cancer cell lines- the uncropped gel of figure 1. Lane 1 and 9. Protein ladder with Myosin (210K) Phosphorylase B (110K), BSA (80K), Ovalbumin (47K), and Carbonic Anhydrase (32K). Lane 2. PC3-5V metastatic prostate clonal cells with empty vector, Lane 3. PrEC-31 transfected with 40 nM of scrambled siRNA. Lane 4 and 5. PrEC-31 transfected with 30 nM and 40 nM of CD82 siRNA, respectively. Lane 6. empty. Lane 7 and 8. PC3-29 and PC3-57 clonal cells restored with CD82, respectively.
